# A call to improve paediatric palliative care quality through research

**DOI:** 10.1186/s12904-023-01262-w

**Published:** 2023-09-26

**Authors:** Laure Dombrecht, Ana Lacerda, Joanne Wolfe, Jennifer Snaman

**Affiliations:** 1 End-of-Life Care Research Group, Vrije Universiteit Brussel and Ghent University, Brussels, Belgium; 2 Portuguese Institute of Oncology, Lisbon Centre, Lisbon, Portugal; 3 Massachusetts General Hospital, Boston, USA; 4 Dana-Farber Cancer Institute, Boston, USA

## Abstract

Paediatric palliative care is needed now more than ever. Medical and technological advances mean that children with complex chronic conditions are surviving longer, necessitating longitudinal support from communities and healthcare systems. Efforts need to be made to ensure our healthcare systems and workforce are equipped to meet the needs of this growing population, including gathering data on the effect of many of our primary and specialty palliative care interventions. *BMC Palliative Care* has launched a new article Collection on “Paediatric palliative care” to provide an open-access resource for all interested in this topic.

Over 20 million children worldwide may benefit from palliative care, yet we currently lack the capacity to provide high quality palliative care to all in need [1]. We therefore not only need to train interprofessional clinicians to provide high quality palliative care, we also must advance the field by building the knowledge base through empiric investigation. In contrast to adult palliative care, there is a lack of high quality evidence on the effectiveness of palliative care interventions in children with serious illness and their families. We hope this editorial will serve as a call to action to paediatric palliative care (PPC) investigators to support this emerging evidence base aimed at improving the care and enhancing quality of life in children with serious illness and those that care for them.

PPC represents an holistic, proactive, interprofessional approach to managing suffering related to serious illness in children, stemming prenatally through young adulthood. The main focus of PPC is on improving quality of life, through the prevention and timely assessment and management of distress [2]. Areas of focus include addressing medical problems (symptoms related to the disease or the disease-focused treatments), and all domains of life - psychological, spiritual, social, economical, educational. Accordingly, the key components of effective PPC are symptom control, family support (including support following death), communication, shared decision-making, and advance care planning.

Over the past several decades and driven by advancement in medicine and technology, healthcare systems across the world have experienced a growing number of children and young people (CYP) with serious illness surviving for much longer than before. Many of these children are assisted with medical devices, such as feeding tubes and ventilators, and require significant care support [3]. Taken together, these transformations necessitate new approaches to providing care of children with serious illness and their families. Many PPC programs are developed to fill a dire need in their current practice or hospital ward, without formal research PPC provision, with only limited insight in which components actually contribute to better care [4]. Another major challenge is defining the remit of PPC - which children and families require specialised PPC? Is it possible (or desirable) to set defined eligibility criteria? Novel clinical care models are needed to deliver specialty palliative care to patients with high distress. Additionally, we must prioritise systematic and organised educational efforts to enhance the primary palliative care skills of clinicians caring for all CYP with serious illness. Collaboration with researchers will aid in the selection of appropriate outcome measures to establish the effect of these clinical and educational initiatives.

In addition to research focused on the testing of care models and educational initiatives, PPC research should work to include the child voice and perspectives (as able), focus on the care of children with rare medical conditions as well as those populations that are disenfranchised or disadvantaged. These research foci require adequate resources and population sizes, both of which have been historically limited in paediatrics. International collaboration is essential to seek the tools and frameworks that may allow us to accomplish this challenge, while also diminishing regional inequities across the globe. Successful coordinated research groups have been developed for certain paediatric diseases (paediatric oncology) and disciplines (critical care); the Pediatric Palliative Care Research Network (PPCRN) is a nascent North American research group that has successfully completed several multisite clinical trials. PPC researchers should look to these groups as models for development of sustainable research collaboratives.

High quality PPC research requires both adequate funding and rigorous training and mentoring of investigators. In the US, longstanding funding mechanisms have ended and may limit resources for future large scale PPC trials. Additionally, clinical palliative care specialty training does not require research training. Together, these two factors undermine the ability to move our field forward.

In addition to challenges with funding for robust research, other barriers include ethical concerns (children as a “vulnerable population”) and language barriers. For example, North America has one primary language while Europe has 24 different languages. Despite our ever-increasingly global world, with a large number and array of languages and cultures, almost all PPC clinical and research tools are only available and tested in English, which impairs the dissemination and implementation of these tools in clinical practice to the patients that may benefit from them the most.

Finally, across the research field of PPC there is a general lack of effectiveness, implementation, and dissemination studies. PPC is an established field, yet most research is descriptive (case studies), retrospective chart reviews, feasibility studies, and small sample (pilot) studies [5]. Researchers and funding sources should prioritise pragmatic trials as well as those focused on developing collaborative research groups.

Working to find answers to these and similar questions will also help us debate models of care delivery. In a world where healthcare demands keep rising, and where resources (staff, equipment, funding) are not growing at the same rate (if growing at all), finding creative and effective ways to deliver the high quality seamless care that seriously ill CYP and their families deserve is paramount.

*BMC Palliative Care* launched a new article Collection devoted to disseminating PPC research: https://www.biomedcentral.com/collections/PPC. We invite you to submit your studies as we continue to build the evidence base for PPC together.

## Data Availability

Not applicable.

## List of abbreviations

CYP: Children and Young People
PPC: Pediatric Palliative Care
PPCRN: Pediatric Palliative Care Research Network
